# Supplementary material for: A Novel Strategy of US3 Codon De-Optimization for Construction of an Attenuated Pseudorabies Virus against High Virulent Chinese Pseudorabies Virus Variant
Source: Vaccines (Basel). 2023 Jul 27;11(8):1288. doi: 10.3390/vaccines11081288 (PMC10458909; doi:10.3390/vaccines11081288)
Supplement: Supplementary file 1 [file vaccines-11-01288-s001.zip › vaccines-2488747-supplementary.pdf]

Supplementary material

Summary

The supporting information includes 1 **supplementary information** and 4 supplementary table.

Supplementary Information

Supplementary Information S1 Nucleotide sequence of US3-1, US3-2, US3-3 and US3^{deop}-1, US3^{deop}-2, US3^{deop}-3. Nucleotide changes compared to original sequence are highlighted in red.

US3-1

ATGGCCGACGCCGGAATCCCCGACGAGATCCTGTACTCGGACATCAGCGACGACGAGATCATCA
TCGACGGCGACGGCGACAGCAGCGGGGACGAGGACACCGACGATGACGGGGGGCTGACGCGG
CAGGCCGCGTCGCGCATCGCCACGGACCTGGGCTTCGAGGTGCTGCAGCCCCTGCAGTCGGGCT
CGGAGGGCCGCGTCTTCGTGGCCCGCCGGCCCGGCGAGGCGGACACGGTGGTGCTGAAGGTGG
GCCAGAAGCCCTCGACGCTGATGGAGGGCATGCTGCTGAAGCGCCTGGCCCACGATAACGTCAT
GAGCCTGAAGCAG

US3-2

ATGCTCGCCCGGGGCCCGGTGACGTGCCTGGTCCTGCCGCACTTTCGGTGCGATCTGTACAGCTA
CCTGACCATGCGGGACGGGCCGCTGGACATGCGCGACGCCGGGCGCGTGATCCGGTCCGTGCTC
CGCGGGCTCGCCTACCTGCACGGGATGCGCATCATGCACCGCGACGTCAAGGCGGAGAACATCT
TCCTCGAGGACGTGGACACGGTGTGCCTGGGGGACCTCGGGGCCGCGCGCTGCAACGTGGCGG
CGCCCAACTTTTACGGGCTCGCCGGGACCATCGAGACCAACGCCCCCGAGGTGCTCGCGCGCG
ACCGCTACGACACCAAGGTCGACGTCTGGGGTGCGGGGGTGGTGCTCTTCGAGACGCTGGCCTA
CCCCAAGACGATCACCGGCGGGGACGAGCCCGCGATCAACGGGGAG

US3-3

ATGCACCTGATCGACCTCATCCGCGCCCTCGGGGTGCACCCCGAGGAGTTCCCGCCCGACACGC
GCCTCCGGAGCGAGTTCGTCCGGTACGCCGGGACCCATCGCCAGCCGTACACGCAGTACGCGC
GCGTGGCTCGCCTCGGGCTGCCCGAGACGGGGGCTTTCCTGATTTACAAGATGTTGACGTTTGAT
CCCGTCCGCCGCCCTTCCGCTGATGAGATACTCAACTTTGGAATGTGGACCGTATAA

US3^{deop}-1

ATGGCCGATGCCGGAATCCCCGATGAAATCCTGTATTCGGATATCAGCGATGATGAAATCATCA
TCGATGGCGATGGCGATAGCAGCGGGGATGAAGATGATGATGATGGGGGGCTGACGCGGGAA
GCCGCGTCGCGCATCGCCACGGATCTGGGCTTTGAAGTGCTGCAACCCCTGCAATCGGGCTCGG
AAGGCCGCGTCTTTGTGGCCCGCCGGCCCGGCGAAGCGGATACGGTGGTGCTGAAAGTGGGCC
AAAAACCCTCGACGCTGATGGAAGGCATGCTGCTGAAACGCCTGGCCCATGATAATGTCATGA
GCCTGAAACAA

US3^{deop}-2

ATGCTCGCCCGGGGCCCGGTGACGTGTCTGGTCCTGCCGCATTTTCGGTGTGATCTGTATAGCTA
TCTGACCATGCGGGATGGGCCGCTGGATATGCGCGATGCCGGGCGCGTGATCCGGTCCGTGCTC
CGCGGGCTCGCCTATCTGCATGGGATGCGCATCATGCATCGCGATGTCAAAGCGGAAAATATCT
TTCTCGAAGATGTGGATACGGTGTGTCTGGGGGATCTCGGGGCCGCGCGCTGTAATGTGGCGGC
GCCCAATTTTTATGGGCTCGCCGGGACCATCGAAACCAATGCCCCCGAAGTGCTCGCGCGCGAT
CGCTATGATACCAAAGTCGATGTCTGGGGTGCGGGGGTGGTGCTCTTTGAAACGCTGGCCTATC

CCAAAACGATCACCGGCGGGGATGAACCCGCGATCAATGGGGAA

US3^{deop}-3

ATGCATCTGATCGATCTCATCCGCGCCCTCGGGGTGCATCCCGAAGAATTTCCGCCCGATACGC
GCCTCCGGAGCGAATTTGTCCGGTATGCCGGGACCCATCGCCAACCGTATACGCAATATGCGCG
CGTGGCTCGCCTCGGGCTGCCCGAAACGGGGGCTTTTCTGATTTATAAAATGTTGACGTTTGATC
CCGTCCGCCGCCCTTCCGCTGATGAAATACTCAATTTTGGAATGTGGACCGTATAA

Supplementary Tables

Table S1 Data presents the immunogenicity of different virus strains in mice

Virus strain	Doses (TCID50)	Numbers	Challenge	
			Survival	Protection Ratio
$PRV^{\Delta TK\&gE\text{-}US3deop\text{-}1}$	$10^{7.0}$	8	6	75%
$PRV^{\Delta TK\&gE\text{-}US3deop\text{-}2}$	$10^{7.0}$	8	4	50%
$PRV^{\Delta TK\&gE\text{-}US3deop\text{-}3}$	$10^{7.0}$	8	3	37.5%
$PRV^{\Delta TK\&gEAH02}$	$10^{7.0}$	8	6	75%
Control group	/	8	0	0%

Table S2 Data presents the pathogenicity of different virus strains in piglets

Virus strain	Inoculation dose (TCID50)	Inoculation route	Fever frequency (≥40.5℃)	Clinical signs
$PRV^{\Delta TK\&gE\text{-}US3deop\text{-}1}$	$10^{6.0}$	intramuscularly	$0^a/5^b$	2/5
$PRV^{\Delta TK\&gE\text{-}AH02}$	$10^{6.0}$	intramuscularly	3/5	2/5
Control group	/	intramuscularly	0/5	0/5

"a" indicates the number of piglets positive; "b" indicates the number of piglets in the group.

Table S3 Data presents the immunogenicity of different virus strains in piglets

Virus strain	immunizing dose (TCID50)	immunization route	Infection dose (TCID50)	Inoculation route	Fever frequency (≥40.5℃)	Clinical signs
$PRV^{\Delta TK\&gE\text{-}US3deop\text{-}1}$	$10^{5.0}$	intramuscularly	$10^{6.5}$	Nasal drip	$0^a/5^b$	0/5
$PRV^{\Delta TK\&gE\text{-}AH02}$	$10^{5.0}$	intramuscularly	$10^{6.5}$	Nasal drip	0/5	0/5
Control group	/	intramuscularly	$10^{6.5}$	Nasal drip	5/5	5/5

"a" indicates the number of piglets positive; "b" indicates the number of piglets in the group.

Table S4 Determination of virus titers in nasal swabs collected daily in the

challenge-infected piglets

Days post challenge	Titer of excreted virus(lgTCID50/mL)		
	PRV$^{\Delta TK\&gE\text{-}AH02}$	PRV$^{\Delta TK\&gE\text{-}US3deop\text{-}1}$	challenge control
0	0	0	0
1	0	0	3.27 ± 0.44
2	0	0	4.48 ± 0.58
3	0	0	5.26 ± 0.31
4	0	0	5.44 ± 0.43
5	0	0	5.25 ± 0.53
6	0	0	4.65 ± 0.21
7	0	0	4.40
8	0	0	/
9	0	0	/
10	0	0	/
11	0	0	/
12	0	0	/
13	0	0	/
14	0	0	/
